# Modified Neural Network with Hysteresis Operators and Adaptive Learning for Tracking Control of Piezoelectric Stack Actuator

**DOI:** 10.3390/mi17070795

**Published:** 2026-06-29

**Authors:** Yuansheng Chen, Wenwu Yang, Lei Yuan, Shaona Liu, Ruijing Zhang, Xinggan Lu, Wei Chen

**Affiliations:** 1 School of Mechanical Engineering, Yancheng Institute of Technology, Yancheng 224051, China; chenys@njust.edu.cn (Y.C.); 13558561263@163.com (W.Y.); 17834874094@163.com (L.Y.); 18404986821@163.com (R.Z.); 2 School of Energy and Power Engineering, Nanjing University of Science and Technology, Nanjing 210094, China; jiuchenlxg@163.com; 3 Hubei Sanjiang Aerospace Hongfeng Control Co., Ltd., Xiaogan 432000, China; 13734928246@163.com

**Keywords:** piezoelectric stack actuator, Prandtl–Ishlinskii model, adaptive signal processing, neural network model

## Abstract

According to the proposed four-layer modified neural network with adaptive learning, an adaptive learning model is designed, and the Play operator weight function update algorithm, the dead-zone operator weight function update algorithm, and the hybrid model program are studied. The hysteresis nonlinearity of a piezoelectric stack actuator at multiple frequencies was tested separately, and the root mean square error (RMSE) of five control methods, including the without control, classic PI and DZ model, and the four-layer modified neural network with an adaptive learning model, were compared through experimental studies. The experimental results show that compared with the without control condition, the RMSE of the classic PI and DZ model is reduced by 67.98% at a frequency of 1 Hz, which can effectively reduce the hysteresis nonlinearity of the piezoelectric stack actuator and has a good hysteresis compensation effect. Compared with the classic PI and DZ model, under the four-layer modified neural network with an adaptive learning model, the RMSE of the piezoelectric stack actuator is reduced by 15.34% at 1 Hz, and the error can still be reduced by 67.75% even at 10 Hz. Indicating that the four-layer modified neural network with an adaptive learning model still has a good hysteresis compensation effect at a wider frequency band.

## 1. Introduction

**Figure 1:**
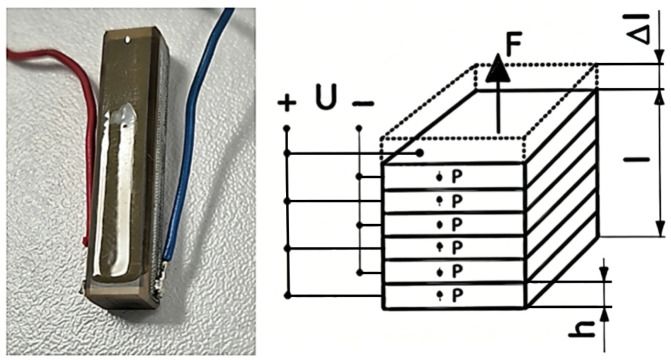
Stacked piezoelectric drivers [11].

As a type of intelligent material, piezoelectric stack actuators have positive and reverse piezoelectric properties [1,2]. The positive piezoelectric properties refers to the phenomenon that when a piezoelectric material is subjected to external physical pressure, the electric dipole moment within the material shortens due to compression, thereby inducing electric polarization. This causes bound charges of opposite signs to appear on the opposing surfaces of the piezoelectric material, and the surface charge density is proportional to the externally applied physical pressure [3,4]. The inverse piezoelectric property refers to the fact that when the piezoelectric material is subjected to an external electric field, the polarization direction of the internal field is consistent with the applied electric field, the material is deformed, and the deformation of the piezoelectric material is directly proportional to the strength of the applied electric field [5,6]. When the applied electric field is an alternating signal, according to its inverse piezoelectric characteristics, the deformation produced by the piezoelectric stack actuator will change with the change of electric field, resulting in vibration [7,8]. At present, according to the relationship between the deformation direction and the axis of piezoelectric ceramics, it can be divided into dual-wafer piezoceramic drivers and piezoelectric stack actuators [9]. The piezoelectric stack actuator is composed of multiple piezoelectric ceramic piezoelectric drives through a physical series [10], an electrically parallel or series connection, and its deformation direction is consistent with the axial direction, so it is also called a telescopic piezoelectric stack actuator. The physical diagrams of piezoelectric stack actuators are shown in Figure 1 [11].

**Figure 2:**
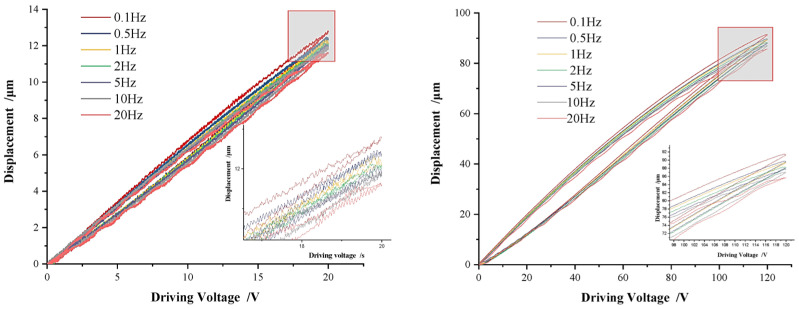
Hysteresis characteristics of a piezoelectric stack actuator [15].

Piezoelectric stack actuators have the advantages of high stiffness, a high displacement resolution, and a small size, so they are often used for micro-positioning devices and precision systems [12]. As a kind of ferroelectric material, the piezoelectric stack actuators exhibit hysteresis nonlinearity in response to the applied voltage [13,14]. When the input voltage changes, the displacement does not follow linearly, but forms a loop. For example, when the voltage rises and falls, the same voltage corresponds to different displacement values, and the displacement curves of the voltage rise and fall stages do not coincide in the displacement–voltage relationship curve, forming a hysteresis loop, in which the hysteresis curve is shown in Figure 2 [15]. In order to reduce the influence of the hysteresis nonlinearity of piezoelectric stack actuators on the precision positioning system, experts mainly modify the hysteresis nonlinearity of piezoelectric stack actuators by modeling the hysteresis nonlinearity and designing the controller in combination with the model [16,17,18]. The mathematical models of the hysteresis nonlinearity of common piezoelectric stack actuators can be divided into two categories: physical models and phenomenal models [19,20]. Most of the mathematical models applicable to a piezoelectric stack actuator are phenomenal models, which do not study the physical nature of hysteresis, but only focus on the relationship between the input voltage and the output displacement of piezoelectric stack actuators [21]. Such models mainly include the Preisach model, Maxwell-Slip model, Duhem model, artificial neural network model, and Prandtl–Ishlinskii (PI) model [16,21,22].

**Figure 3:**
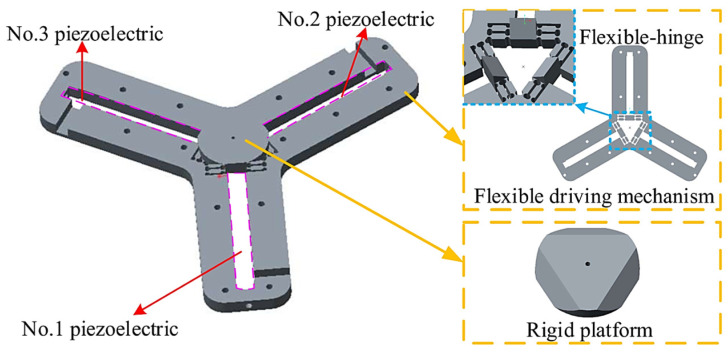
Symmetrical rigid–flexible coupling micro-positioning platform [25].

Due to the limited output displacement of piezoelectric stack actuators, in order to better observe the hysteresis of piezoelectric stack actuators, the positioning platform is usually used to amplify the output displacement of piezoelectric stack actuators [23,24]. The platform is mainly composed of two parts: a flexible drive mechanism and a rigid platform that is shown in Figure 3. The flexible drive mechanism is composed of three groups of symmetrical parallel circular flexure hinge structures, which have a small structural parasitic movement, a strong load-bearing capacity, and small dynamic bad coupling [25]. Meanwhile, a wedge structure is set in the middle of each group of symmetrical parallel flexure hinges, and the wedge angle is 45° [25]. The rigid platform has an inclined plane with the same angle as the wedge for easy placement.

It is difficult to identify the parameters of traditional hysteresis models, but due to the gradual maturity of artificial neural network models, model parameters can be identified through neural networks [26,27,28]. Uralde [29] designed a model that feeds MPC controllers through artificial neural networks, which can simplify the acquisition of models and realize accurate mathematical models of PEA dynamics. Xiong [30] designed a new recurrent neural network of PEA-RNN, which adopts a three-input, one-output approach, and the experimental results show that it can accurately compensate for the dynamic hysteresis nonlinearity of a piezoelectric stack actuator, providing a basis for the online updating and identification of the model in the future with a shorter training cycle. Similarly, Hu [31,32] designed a convolutional neural network model based on the PI model, which consists of a rate-dependent PI model layer and a convolutional network layer, which can describe rate-dependent hysteresis phenomena and improve the generalization ability of hysteresis models, respectively. The experimental results show that it has higher accuracy and a stronger generalization ability compared to the classical PI model.

The classical Prandtl–Ishlinskii hysteresis model is proposed, and the model is modified. A dead-zone operator is added to the classical PI model to establish and modify the Prandtl–Ishlinskii model. Then, on the basis of the modified Prandtl–Ishlinskii model, a neural network model is combined with it, and a four-layer modified neural network with an adaptive learning model is proposed.

## 2. Modeling the Hysteresis of Piezoelectric Stack Actuator

The classical Prandtl–Ishlinskii hysteresis model is a modified version of the Preisach model. The Play hysteresis operator is established as the hysteresis unit, and the hysteresis relationship of the piezoelectric stack actuators is described by the mathematical relationship between the hysteresis operators [14]. The establishment of the classical PI model does not need to understand the physical mechanism of hysteresis between the input voltage and the output displacement of the piezoelectric stack actuator, which makes the model structure simpler, the amount of data calculation is smaller, and the hysteresis modeling process is simplified [15,16].

### 2.1. Prandtl–Ishlinskii Hysteresis Model

**Figure 4:**
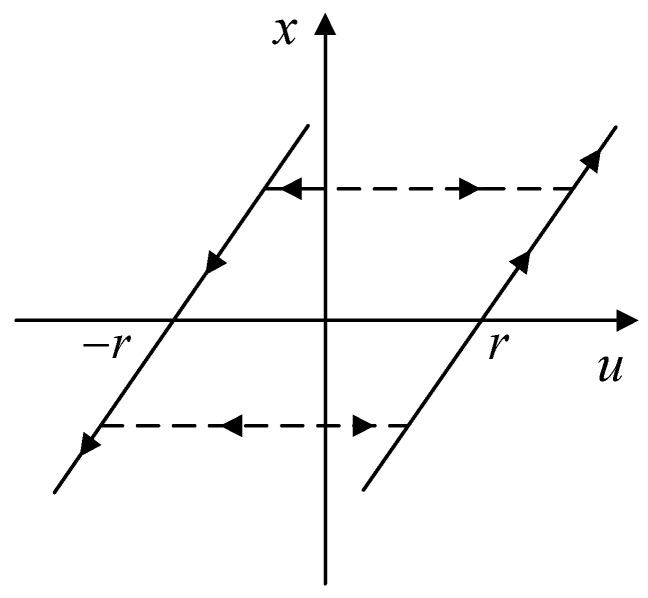
Play operator input and output diagram.

The classical PI model describes the hysteresis characteristics of the system by weighting the Play operator (or backlash operator) with different weights and thresholds, which originally described the nonlinear characteristics of gear clearance in the transmission systems. Thus, it can be seen that the Play operator is the basic unit that constitutes the PI hysteresis model, and this operator is a continuous and symmetrical rate-independent operator. Its basic definition is as follows:

Suppose $C_{m}\left[0,t_{E}\right]$ is a set of segmented and monotonic continuous functions, when an arbitrary input signal $u\left(t\right)\in C_{m}\left[0,t_{E}\right]$ is applied, dividing the time domain into N sub-intervals $0=t_{0}<t_{1}<\cdots <t_{N-1}<t_{N}=t_{E}$, and satisfy that the input signal $u\left(t\right)$ is monotonically continuous in the interval $\left[t_{i},t_{i+1}\right],i=0,1,2,\cdots N-1$. The input and output relationship of the Play operator is shown in Figure 4.

Its analytical equation can be expressed as:

(1) $$v\left(t\right)=H_{r}\left[u,v_{0}\right]\left(t\right)\;\;\;\;\;\;\;\;\;\;\;\;\;=\max\left\{u\left(t\right)-r,\min\left\{u\left(t\right)+r,v_{0}\left(t_{i}\right)\right\}\right\}\;\;\;\;\;\;\;\;\;\;t_{i}<t<t_{i+1},i=0,1,2,\cdots ,N-2.N-1$$

where $u\left(t\right)$ is the operator input signal, $v\left(t\right)\;$ is the operator output signal, and r is the operator threshold $\left(r\geq 0\right)$.

The hysteresis characteristics of the Play operator are mainly related to the threshold r, and the value of the threshold r determines the hysteresis nonlinearity of the Play operator. Therefore, the reasonable selection of the number of Play operators and the value of the threshold can effectively improve the accuracy of modeling. The threshold of the Play operator is determined by the range of the input signal $u\left(t\right)$.

(2) $$r_{i}=\frac{i}{n}(\max\{u(t)-\min\{u(t)\}\},i=0,1,2,\cdots ,n-1$$

**Figure 5:**
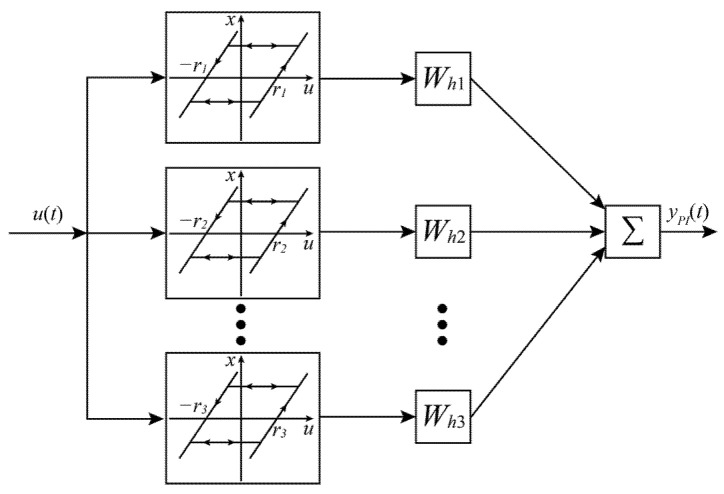
Schematic diagram of the PI model structure.

The classical PI model describes the hysteresis characteristics of the system by the weighted superposition of the play operator, and the model structure is shown in Figure 5.

Figure 5 shows the classical PI hysteresis model that can be obtained by integrating the product of the Play operator and its weight function on the threshold interval [0, $r_{max}$] and its analytical equation is as follows:

(3) $$y_{PI}\left(t\right)=\int_{0}^{r_{max}}W_{H}\left(r\right)H_{r}\left[u,v_{0}\right]\left(t\right)dr$$

where $y_{PI}\left(t\right)$ is the output of the PI model, $W_{H}\left(r\right)$ is the computation of the weight value with respect to the threshold r, $r_{max}$ is the maximum threshold which is determined by Equation (2).

For simplicity in practical modeling and calculation, the PI model needs to be discretized. The integral of the Play operator over the threshold interval is discretized into a weighted superposition of several hysteresis operators within the threshold interval. Therefore, the output of the classical PI model after discretization of Equation (3) is as follows:

(4) $$y_{PI}\left(t\right)=\sum_{i}^{n}W_{Hi}H_{ri}\left[u,v_{0}\right]\left(t\right),i=0,1,2,\cdots n$$

where $W_{H}$ is the weight vector of the Play operator, and $H_{r}\left[u,v_{0}\right]\left(t\right)$ is the vector of the Play operator.

(5) $$W_{H}^{T}=\left[w_{h1},w_{h2}\cdots ,w_{hm}\right]$$

(6) $$H_{r}\left[u,v_{0}\right]\left(t\right)=\left[\begin{array}{c} \max\left\{u\left(t\right)-r_{1},\min\left\{u\left(t\right)+r_{1},v_{01}\left(t_{i}\right)\right\}\right\}\\ \max\left\{u\left(t\right)-r_{2},\min\left\{u\left(t\right)+r_{2},v_{02}\left(t_{i}\right)\right\}\right\}\\ \vdots \\ \max\left\{u\left(t\right)-r_{m},\min\left\{u\left(t\right)+r_{m},v_{0m}\left(t_{i}\right)\right\}\right\} \end{array}\right]$$

$$t_{i}<t<t_{i+1},i=0,1,2,\cdots ,N-2.N-1$$

The vector expression of the discretized PI model is as follows:

(7) $$y_{PI}\left(t\right)={W_{H}}^{T}H_{r}\left[u,v_{0}\right]\left(t\right)\;\;\;\;\;\;\;\;\;\;\;\;\;\;\;\;\;\;\;\;=\left[w_{h1},w_{h2}\cdots ,w_{hm}\right]\left[\begin{array}{c} \max\left\{u\left(t\right)-r_{1},\min\left\{u\left(t\right)+r_{1},v_{01}\left(t_{i}\right)\right\}\right\}\\ \max\left\{u\left(t\right)-r_{2},\min\left\{u\left(t\right)+r_{2},v_{02}\left(t_{i}\right)\right\}\right\}\\ \vdots \\ \max\left\{u\left(t\right)-r_{m},\min\left\{u\left(t\right)+r_{m},v_{0m}\left(t_{i}\right)\right\}\right\} \end{array}\right]$$

where m is the number of the Play operator, and r is the threshold of the Play operator.

By observing the expressions of the classical PI model, it is not difficult to find that the classical PI model is composed of two parts, one is the linear function of the input signal $u\left(t\right)$, and the other is the weighted superposition of the play operator. It can be seen from Figure 5, the input–output relationship of the Play operator is centrally symmetrical. Therefore, the hysteresis curve described by the classical PI model is also symmetrical. Consequently, the hysteresis curve described by the classical PI model is always counterclockwise and odd-symmetric.

### 2.2. Dead-Zone Operator

A variety of modified methods for PI model operators are proposed for different application situations, and the special modeling method used in this study is very close to the complex PI nonlinear model, and is largely consistent with the classical PI model [17,18]. It is obtained by weighted superposition of one-sided dead-zone operators based on different thresholds. The output expression of the classic dead-zone operator is as follows:

(8) $$v\left(t\right)=S_{R_{D}}\left[u\right]\left(t\right)=S\left[u,R_{D}\right]\left(t\right)$$

where $v\left(t\right)$ is the output of the classic dead-zone operator, $u\left(t\right)$ is the input of the classic dead-zone operator, and $R_{D}$ is the threshold value of the classic dead-zone operator.

In order to understand the one-sided classical dead-zone operator more accurately, the analytic equation can be expressed as follows:

(9) $$S\left(u,R_{D}\right)\left(t\right)=\left\{\begin{array}{c} \max\{u(t)-R_{D},0\};\\ u\left(t\right);\\ \min\{u(t)-R_{D},0\}; \end{array}\right.\begin{array}{c} R_{D}>0\\ R_{D}=0\\ R_{D}<0 \end{array}$$

**Figure 6:**
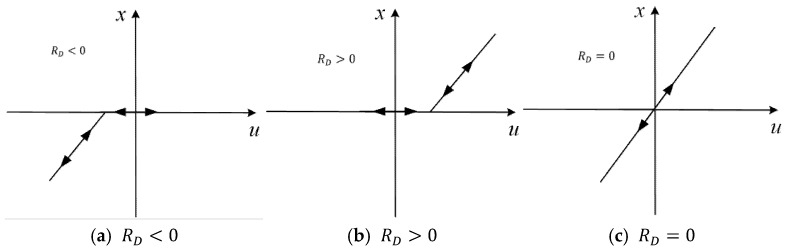
Diagram of the input–output relationship of the classic dead-zone operator.

From the above equation, the output of the classical dead-zone operator is completely determined by the threshold $R_{D}$. Figure 6 shows the input–output relationship of the classic dead-zone operator at different thresholds.

Therefore, the classical dead-zone operator is a continuous, nonlinear, rate-independent operator.

(10) $$y_{DZ}\left(t\right)=\int_{-\infty }^{+\infty }W_{D}\left(R_{D}\right)S\left[u,R_{D}\right]\left(t\right)dR_{D}$$

The output expression is:

(11) $$y_{DZ}\left(t\right)=W_{D}^{T}\cdot S_{R_{D}}\left[u\right]{\left(t\right)}^{T}$$

where $W_{D}^{T}$ is the weight of the classic dead-zone operator, $R_{D}$ is the threshold value of the classic dead-zone operator, and the vector expression is as follows:

(12) $$W_{D}^{T}=\left[\begin{array}{cccc} \begin{array}{cccc} W_{D-l} & \cdots & W_{D-1} & W_{D0} \end{array} & W_{D1} & \cdots & W_{Dl} \end{array}\right]$$

$$S_{R_{D}}\left[u\right]{\left(t\right)}^{T}=[S_{R_{D-l}}\left[u\right](t),\cdots ,S_{R_{D-1}}\left[u\right](t),S_{R_{D0}}\left[u\right](t),S_{R_{D1}}\left[u\right](t),\cdots ,S_{R_{Dl}}\left[u\right](t)]$$

(13) $$-\infty <\cdots <R_{D-1}<\cdots <R_{D-1}<R_{D0}=0<R_{D1}<\cdots <R_{Dl}<\cdots <+\infty$$

### 2.3. Modified Prandtl–Ishlinskii Model

The positive linear transfer function in the neural network is different from the classical dead-zone operator.

(14) $$y_{PIDZ}\left(t\right)=\int_{R_{Dmin}}^{R_{Dmax}}\int_{0}^{r\_max}W_{D}{\left(R_{D}\right)S[W}_{H}\left(r\right)H_{r}\left[u,v_{0}\right],R_{D}](t)drdR_{D}$$

The modified Prandtl–Ishlinskii model can be described as:

(15) $$y_{PIDZ}\left(t\right)=W_{D}^{T}S_{R_{D}}\left[y_{PI}\right]\left(t\right)$$

(16) $$y_{PI}\left(t\right)=\sum_{i=1}^{n}W_{Hi}y_{i}=W_{H}^{T}H_{r}\left[u\left(t\right),v_{0}\right]$$

(17) $$S_{R_{D}}\left[y_{PI}\right]\left(t\right)=S_{R_{D}}W_{H}^{T}H_{r}\left[u\left(t\right),v_{0}\right]$$

Substitute Equation (17) into (15):

(18) $$y_{PIDZ}\left(t\right)=W_{D}^{T}S_{R_{D}}W_{H}^{T}H_{r}\left[u\left(t\right),v_{0}\right]$$

$y_{PIDZ}\left(t\right)$ is the modified output of the Prandtl–Ishlinskii model for k-moment, $S_{R_{D}}\left[y_{PI}\right]\left(t\right)$ is the output vector of the dead-zone operator at time t, $W_{D}$ is the weight vector of the dead-zone operator, $W_{D_{j}}\;$ is the weight of the *j*-th dead-zone operator, and $R_{D}$ is the threshold of the dead-zone operator.

Considering the input of a piezoelectric stack actuator is the forward voltage, the threshold selection of the dead-zone operator is as follows:

(19) $$R_{D0}=0<R_{D1}<\cdots <r_{Dl}<\cdots <+\infty$$

## 3. Four-Layer Modified Neural Network with an Adaptive Learning Model

### 3.1. Four-Layer Modified Neural Network Model with Hysteresis Modeling

**Figure 7:**
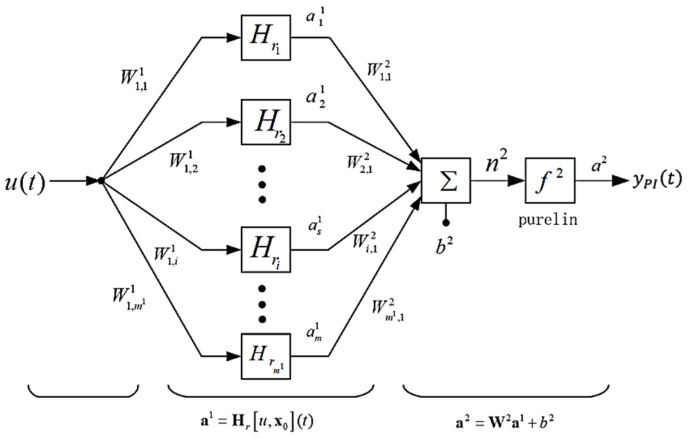
Topology diagram of the neural network of the classical PI model.

It is known that neural network models can theoretically be capable of fitting any nonlinear function with arbitrary precision [19]. However, in the actual modeling, it is found that a neural network cannot describe the hysteresis nonlinear characteristics of a piezoelectric stack actuator, so the four-layer modified neural network model is proposed [20,21]. In order to better compare the structure diagram of the neural network model and the classical PI model, the structural diagram of the classical PI model is simplified, as shown in Figure 7.

It is not difficult to find that the structure is similar to the neural network model, so the classical PI model can be regarded as a two-layer neural network model with only one input signal. The weight matrix of the first-layer input signal is:

(20) $$W^{1}=\left[\begin{array}{c} w_{1,1}^{1}\\ w_{1,2}^{1}\\ \vdots \\ w_{1,m^{1}}^{1} \end{array}\right]$$

If the weight matrix is $W^{1}$ (1 is represented as the first layer of the neural network) and each element has a weight of 1, then the output vector of the first-layer neural network model is:

(21) $$a^{1}=\left[\begin{array}{c} a_{1}^{1}\\ a_{2}^{1}\\ \vdots \\ a_{m^{1}}^{1} \end{array}\right]=W^{1}H_{r}\left[u,v_{0}\right]$$

$$w_{1,i}^{1}=1,i=1,2,3,\cdots ,m$$

If the second-layer neural network model consists of a neuron with a pure linear function, then the output vector of the second-layer neural network is:

(22) $$a^{2}=f^{2}\left(n^{2}\right)=n^{2}\;\;\;\;\;\;\;\;\;=W^{2}a^{1}+b^{2}$$

**Figure 8:**
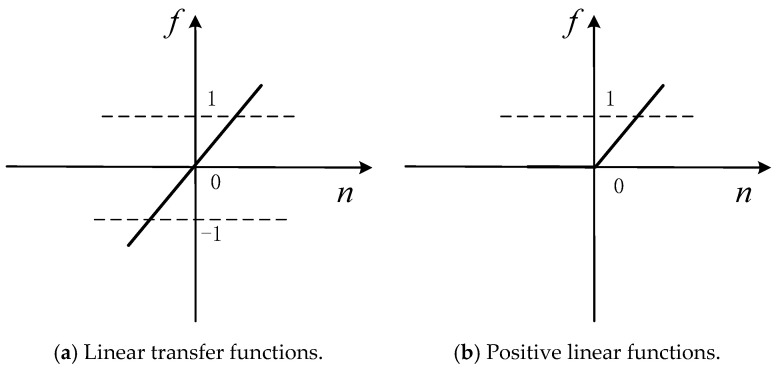
Schematic diagram of the transfer function.

$f^{2}$ is a linear transfer function, this is shown in Figure 8a. $b^{2}$ is the offset value of the second layer of neurons.

Suppose the offset value is $b^{2}=0$, $W^{2}=W_{H}$, then the value of the output vector of the second-layer neural network $a^{2}$ is equal to the output value of the classical PI model $y_{PI}\left(t\right)$.

(23) $$a^{2}=W_{H}a^{1}\;\;\;\;\;\;\;={W_{H}H}_{r}\left[u,v_{0}\right]$$

Therefore, the two-layer neural network model is equivalent to the classical PI model.

In the context of deep learning, linear transfer functions usually refer to identity mapping relationships.

The positive linear functions is:

(24) $$z\left(x\right)=poslin\left(x\right)=\max\left(0,x\right)$$

$$\;\;\;\;\;\;a=poslin\left(n\right)=\max\left(0,n\right)$$

where n is the input of the upper layer of the neural network, and the output of the neural network is:

(25) $$a=poslin\left(0,pw+b\right)$$

When the offset value is $b=-R_{D}$, w=1, $p=u\left(t\right)$:

(26) $$a=\max\left\{u\left(t\right)-R_{D},0\right\}\;R_{D}>0$$

At this time, the positive linear function is consistent with the DZ operator. Therefore, the neural network method can be used to identify the model parameters.

**Figure 9:**
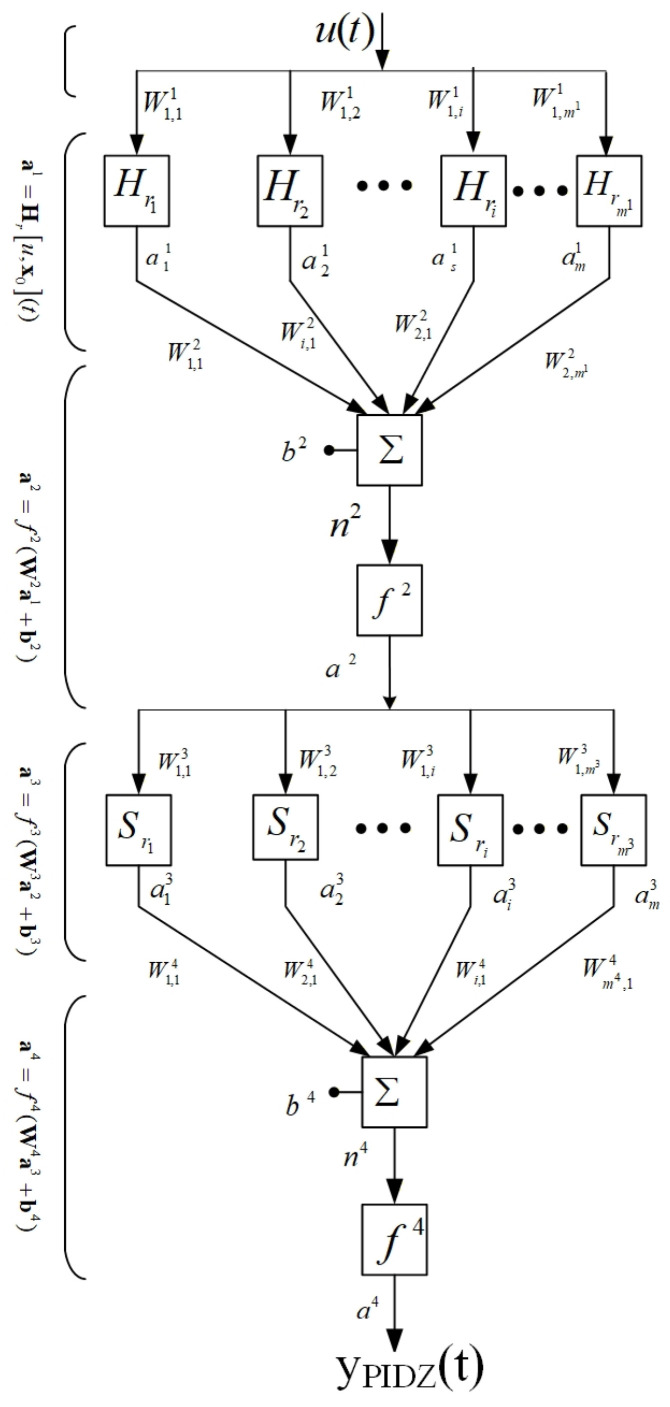
Diagram of the topology of the four-layer modified neural network model.

The comparison between the classical neural network model and the classical PI model shows that they are equivalent models. Therefore, the modified Prandtl–Ishlinskii model can also be equivalent to the four-layer neural network model. The structural diagram is shown in Figure 9.

In Figure 9, the output of the first-layer neural network model $a^{1}$ and the second-layer neural network model $a^{2}$ have both been obtained; the output of the second-layer neural network serves as the input to the third-layer neural network model. Among them, the weight matrix of the third-layer neural network is:

(27) $$W^{3}=\left[\begin{array}{c} w_{1,1}^{3}\\ w_{1,1}^{3}\\ \vdots \\ w_{1,m^{3}}^{3} \end{array}\right]$$

Then, the output vector of the third-layer neural network is:

(28) $$a^{3}=\left[\begin{array}{c} a_{1}^{3}\\ a_{2}^{3}\\ \vdots \\ a_{m^{3}}^{3} \end{array}\right]=f^{3}\left[n_{j}^{3}\right]=\left\{\begin{array}{c} 0{,\;\;n}_{j}^{3}<0\\ n_{j}^{3},\;\;n_{j}^{3}\geq 0 \end{array}\right.$$

(29) $$n_{j}^{3}=\sum_{i=1}^{m^{3}}w_{i}^{3}a_{i}^{2}+b_{j}^{3}$$

$$\;\;{\;w}_{1i}^{3}=1,i=1,2,3,\cdots ,m^{3}$$

$b^{3}$ is the third-layer neural network offset value. $f^{3}$ is a positive linear function, as shown in Figure 8b.

(30) $$f^{3}=f\left(poslin\right)=S_{r_{s}}$$

The output vector of the fourth-layer neural network is:

(31) $$a^{4}=f^{4}\left[n^{4}\right]=W^{4}a^{3}+b^{4}$$

And $b^{4}$ is the fourth-layer neural network offset value. $f^{4}$ is a positive linear function, as shown in Figure 8a.

Suppose the offset value is $b^{4}=0$, $W^{4}=W_{D}$.

(32) $${a_{j}}^{4}=W_{Dj}a^{3}=W_{Dj}f^{3}\left[n_{j}^{3}\right]=\left\{\begin{array}{c} 0,\;\;n_{j}^{3}<0\\ {W_{Dj}n}_{j}^{3},\;\;n_{j}^{3}\geq 0 \end{array}\right.$$

In the case of $b^{2}=0$ where the weight vector of the second-layer neural network $W^{2}$ and the weight function of the classical PI model $W_{H}$ are equal, a neuron in the first and second layers of the neural network forms a Play operator. In the second layer of the neural network $m^{1}$ neurons can be found, Therefore, the modified neural network model has $m^{1}$ Play operators. At the same time, it is assumed that the weight vector of the fourth-layer neural network $W^{4}$ and the weight vector $W_{D}$ of the dead-zone operator are equal, and $b^{4}=0$; in this case, a neuron in the third-layer neural network and the fourth-layer neural network forms a dead-zone operator. In the third-layer of the neural network an $m^{3}$ neuron can be found; therefore, the four-layer modified neural network model has $m^{3}$ dead-zone operators. Therefore, the four-layer modified neural network model can describe both the asymmetry and nonlinear characteristics of a piezoelectric stack actuator.

### 3.2. Adaptive Learning

Firstly, a hysteresis control model is established for the hysteresis nonlinearity phenomenon of a piezoelectric stack actuator, and the inverse model is calculated on this basis. Secondly, the inverse model is connected in a series before the piezoelectric stack actuator to form a linearized system. Ideally, the expected input displacement is equal to the output measurement displacement, but there are errors in the external environment due to noise, vibration, and other uncertainties:

(33) $$e\left(t\right)=X_{i}-X_{o}$$

$X_{i}$ is the expected input displacement, and $X_{o}$ is the output measurement displacement.

For online parameter identification, the last layer equation for sensitivity backpropagation in neural networks is introduced:

(34) $$s^{M}=-2{\dot{F}}^{M}\left(n^{M}\right)e\left(t\right)$$

$s^{M}$ is the sensitivity of the M layer of the neural network, and its backpropagation method is $s^{M}\to s^{M-1}\to \cdots \to s^{2}\to s^{1}$, $e\left(t\right)$ is the error signal of the piezoelectric stack actuator to measure the displacement and the desired displacement, and ${\dot{F}}^{M}\left(n^{M}\right)$ is the output matrix of the neural network, which is specifically defined as:

(35) $${\dot{F}}^{M}\left(n^{M}\right)=\left[\begin{array}{cccc} {\dot{f}}^{m}(n_{1}^{m}) & 0 & \cdots & 0\\ 0 & {\dot{f}}^{m}(n_{2}^{m}) & \cdots & 0\\ \vdots & \vdots & & 0\\ 0 & 0 & \cdots & {\dot{f}}^{m}(n_{s^{m}}^{m}) \end{array}\right]$$

According to chain law in the form of Jacobi matrices, we can know that the recursive equation for sensitivity is:

(36) $$s^{m}=\frac{\partial \hat{F}}{\partial n^{m}}={\left(\frac{\partial n^{m+1}}{\partial n^{m}}\right)}^{T}\frac{\partial \hat{F}}{\partial n^{m+1}}={\dot{F}}^{M}\left(n^{M}\right){\left(W^{m+1}\right)}^{T}\frac{\partial \hat{F}}{\partial n^{m+1}}\;\;\;\;={\dot{F}}^{M}(n^{M}){\left(W^{m+1}\right)}^{T}s^{m+1},\;m=M-1,\cdots ,2,1$$

Finally, the classical PI model and the dead-zone operator weights are updated using the approximate fastest descent method, which is in Equation (37) as:

(37) $$W^{m}(k+1)=W^{m}(k)-\alpha s^{m}(a^{m-1}{)}^{T}$$

where k is the number of model iterations, α is the model learning rate, $W^{m}$ is the weight vector of the model layer m, and $a^{m-1}$ is the output vector of the m−1 layer of the model.

The fourth layer sensitivity of the four-layer modified neural network with an adaptive learning model can be calculated by Equations (34) and (35):

(38) $$s^{4}=-2e\left(t\right)$$

The third layer of sensitivity based on the four-layer modified neural network with an adaptive learning model can be obtained by the recursive Equation (36) for sensitivity:

(39) $$s^{3}={\dot{F}}^{3}\left(n^{3}\right){\left(W^{4}\right)}^{T}s^{4}$$

(40) $${\dot{F}}^{3}\left(n^{3}\right)=\left[\begin{array}{cccc} \frac{\partial S_{r_{1}}}{\partial n_{1}^{3}} & 0 & \cdots & 0\\ 0 & \frac{\partial S_{r_{2}}}{\partial n_{2}^{3}} & \cdots & 0\\ \vdots & \vdots & & 0\\ 0 & 0 & \cdots & \frac{\partial S_{r_{m}}}{\partial n_{m}^{3}} \end{array}\right]$$

$\frac{\partial S_{r_{m}}}{\partial n_{m}^{3}}$ is the derivative of the positive linear function:

(41) $$\frac{\partial S_{r_{m}}}{\partial n_{m}^{3}}=\left\{\begin{array}{c} \begin{array}{cc} 1, & n_{i}^{3}>R_{D} \end{array}\\ \begin{array}{cc} 0, & n_{i}^{3}\leq R_{D} \end{array} \end{array}\right.$$

Substituting Equation (40) into Equation (39) gives the third level of sensitivity $s^{3}$:

(42) $$s^{3}=\left\{\begin{array}{cc} -2e\left(t\right){\left(W^{4}\right)}^{T}, & n_{i}^{3}>R_{D}\\ 0, & n_{i}^{3}\leq R_{D} \end{array}\right.$$

Then, according to the sensitivity recursive equation, the second layer sensitivity based on the four-layer modified neural network with an adaptive learning model is obtained:

(43) $$s^{2}={\dot{F}}^{2}\left(n^{2}\right){\left(W^{3}\right)}^{T}s^{3}=\frac{\partial f^{2}}{\partial n^{2}}{\left(W^{3}\right)}^{T}s^{3}$$

$f^{2}$ is the linear transfer function of the second-layer neural network, that is $\frac{\partial f^{2}}{\partial n^{2}}=1$, $W^{3}$ is the weight vector of the third-layer neural network, which needs to initialize the connection weights between the input layer, hidden layer, and output layer directly in the model training. Therefore, $W^{3}$ assumes that each element in the supposed network has a weight of 1, $W^{3}=\left[1,1,\cdots ,1\right]$. The sensitivity of the second layer of neural network can be obtained as:

(44) $$s^{2}=s^{3}=\left\{\begin{array}{cc} -2e\left(t\right){\left(W^{4}\right)}^{T}, & n_{i}^{3}>R_{D}\\ 0, & n_{i}^{3}\leq R_{D} \end{array}\right.$$

Finally, according to Equation (37), the weight of the second layer neural network can be calculated, and the updated equation is:

(45) $$W^{4}\left(k+1\right)=W^{4}\left(k\right)+\alpha_{S}2e\left(t\right){\left(a^{3}\right)}^{T}$$

(46) $$W^{2}\left(k+1\right)=W^{2}\left(k\right)+\alpha_{H}s^{2}{\left(a^{1}\right)}^{T}$$

$\alpha_{S}$ is the learning rate of the dead-zone operator model, and $W^{4}\left(k+1\right)$ is the updated weight vector of the dead-zone operator model. $\alpha_{H}$ is the learning rate of the classical PI model, and $W^{2}\left(k+1\right)$ is the updated weight vector of the classical PI model.

## 4. Tracking Experiments

### 4.1. Experimental Platform Construction

**Figure 10:**
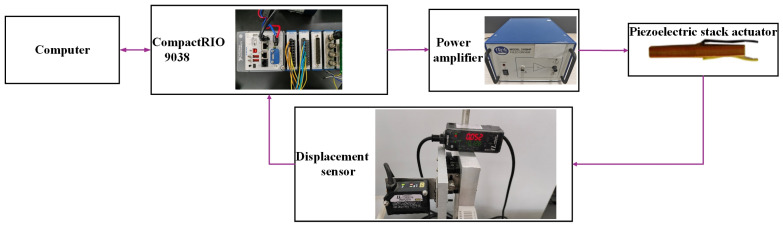
Experimental setup.

The overall design principle of the system is to adopt the modular design concept, considering the scalability and upgradeability of the equipment. In terms of compilation programs, LabVIEW, and LabVIEW-Real-Time modules work together to develop the CompactRIO system. The system consists primarily of a Computer and a CompactRIO embedded system. Among them, the Computer is mainly used as a human–computer interaction interface, configuring synchronization system parameters, data communication, and receiving and displaying data. The CompactRIO embedded system consists of an embedded controller, a digital I/O module with corresponding functions, and a synchronous analog signal input module. The framework of the system is shown in Figure 10.

**Figure 11:**
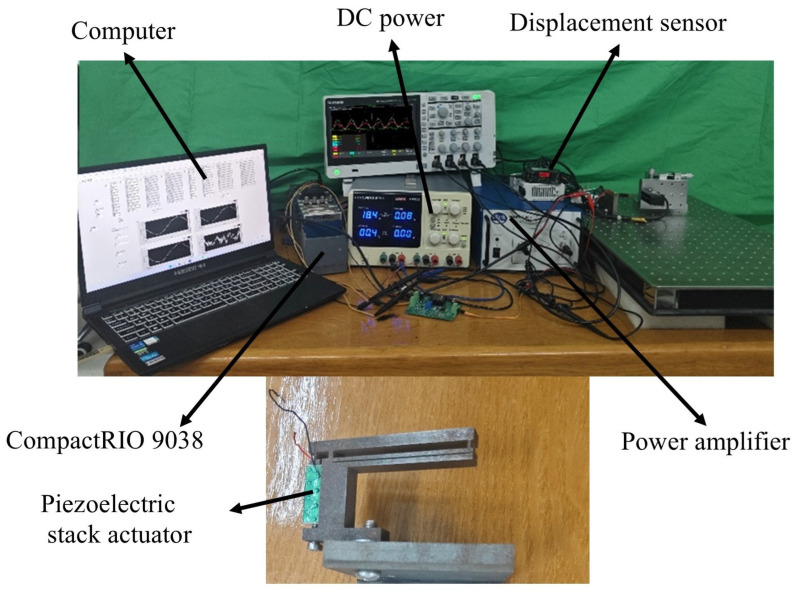
Experimental platform for piezoelectric stack actuator.

The overall experimental platform for piezoelectric stack actuator power supply is shown in Figure 11.

(1) Displacement sensors and piezoelectric stack actuator

The main function of the displacement sensor in the experiment is to convert the displacement signal of the piezoelectric stack actuator into a voltage signal, and then transmit it to the modified neural network model established by LabVIEW through the acquisition of the embedded controller CompactRIO 9038, which is used to subtract the error signal from the expected displacement signal to obtain the compensated voltage signal. The resolution of the displacement sensor (Type IL-S025, Keyence (China) Co., Ltd., Shanghai, China) is 1 μm, and its sensitivity is 0.5 V/mm. The piezoelectric stack actuator was selected for the bar actuator (5 mm × 5 mm × 36 mm). The stroke of the piezoelectric stack actuator (from Sinocera Piezotronics, Inc., Yangzhou, China) is 28 μm at 150 V. The elongation of the piezoelectric stack actuator is amplified by a micro-gripper structure according to the lever principle.

The piezoelectric stack actuator and the displacement sensor are fixed on the shock-absorbing platform, and the spiral micrometer is adjusted so that the vertical distance between the displacement sensor and the piezoelectric stack actuator is within the effective measurement range of the sensor, and then the displacement sensor is zeroed. The physical diagram of the displacement sensor and piezoelectric stack actuator is shown in Figure 11.

(2) CompactRIO 9038 Embedded Control System

In the experiment, CompactRIO 9038 is selected as the main control module for software design, and its main function is to transmit the measurement signal generated by the displacement sensor to the modified neural network model through its acquisition board NI 9215, and then output the analog voltage signal after hysteresis compensation through its output board NI 9263. The computer with installed LabVIEW 2025 implement in control algorithm on CompactRIO 9038.

### 4.2. Hysteresis Compensation Experimental Verification

**Figure 12:**
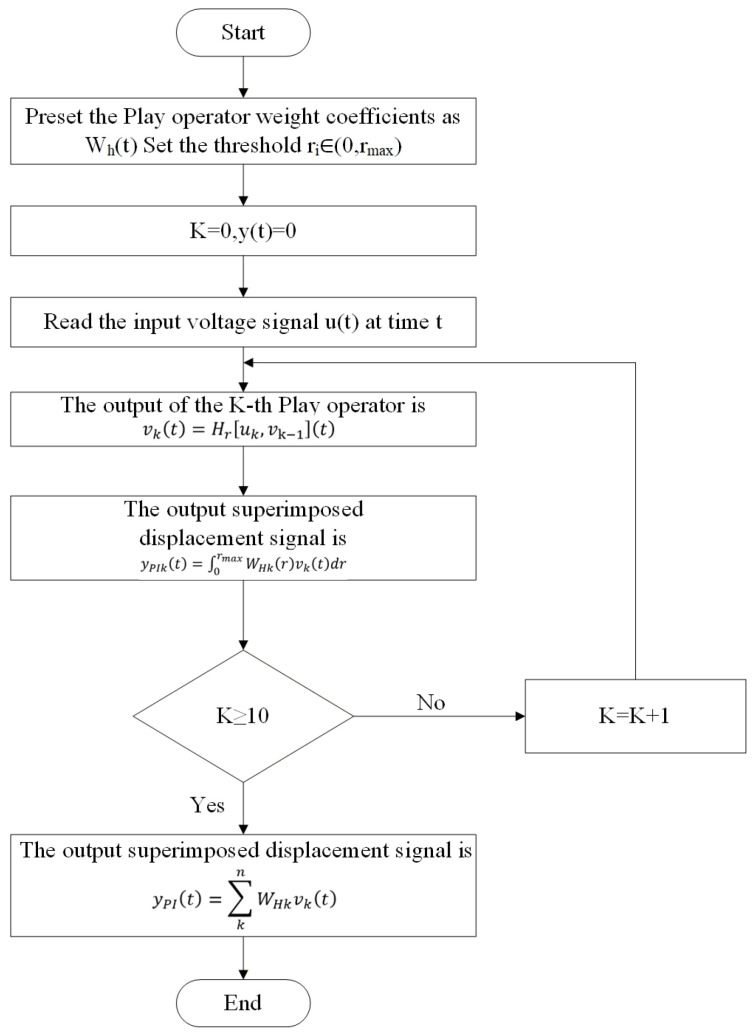
Flowchart of the Play operator program.

Eleven Play operators are selected, and their thresholds are [0, 0.05, 0.1, ⋯, 0.45, 0.5]. The output of each operator is calculated according to the equation, and then the 11 Play operators are weighted and superimposed to obtain the output of the classical dead-zone model. The building of a classic PI model on LabVIEW is shown in Figure 12.

**Figure 13:**
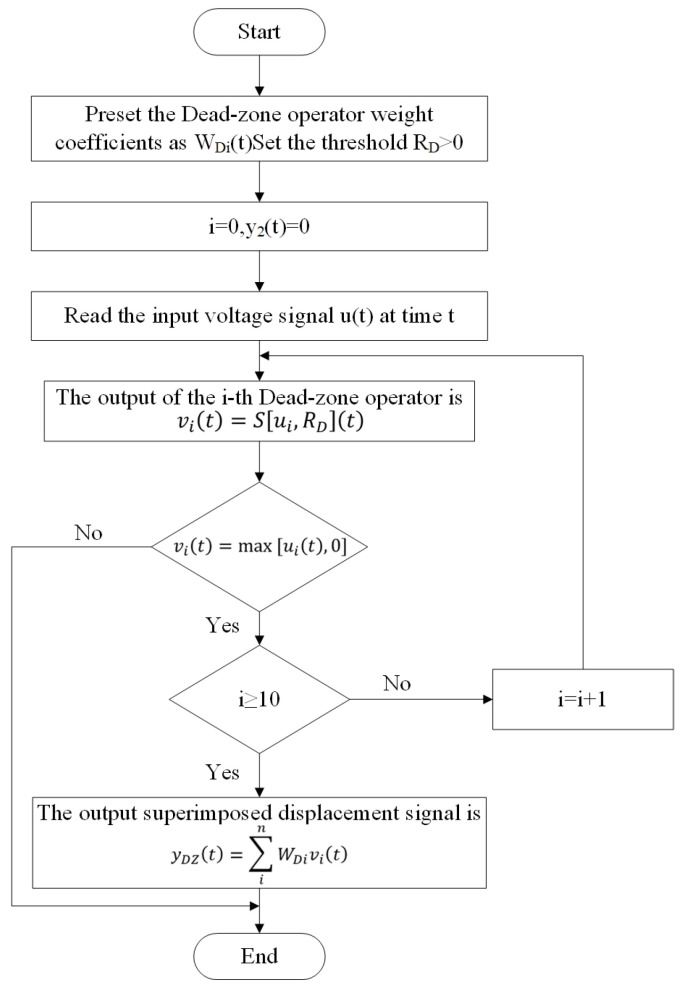
Flowchart of the dead-zone operator program.

Ten dead-zone operators are selected, and their thresholds are [0, 0.1, 0.2, ⋯, 0.8, 0.9]. The output of each operator is calculated according to the equation, and then the 10 dead-zone operators are weighted and superimposed to obtain the output of the dead-zone operator model. The model building procedure on LabVIEW is shown in Figure 13.

By combining the Play operator and the dead-zone operator program diagram, the final modified Prandtl–Ishlinskii model can be obtained, which can accurately describe the asymmetric and nonlinear characteristics of a piezoelectric stack actuator.

**Figure 14:**
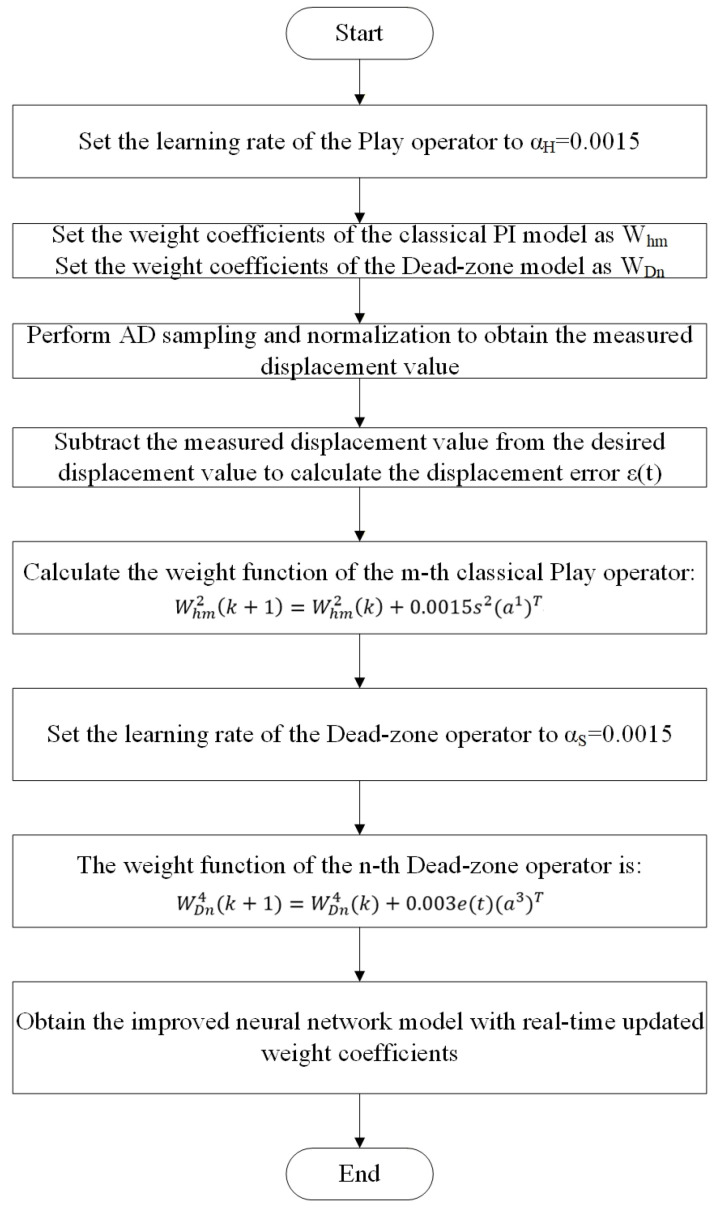
Flowchart of the adaptive inverse control program.

The error signal of the measured displacement and the desired displacement of the piezoelectric stack actuator is selected as the input signal of the adaptive learning algorithm, and the output signal is obtained as the weight vector of the four-layer modified neural network with an adaptive learning model. The weight vector of the four-layer modified neural network with an adaptive learning model is updated through adaptive learning, thereby reducing the error caused by hysteresis modeling. So that the four-layer modified neural network with an adaptive learning model can better eliminate the hysteresis nonlinearity phenomenon of a piezoelectric stack actuator, and then reduce the system error signal. The specific adaptive learning procedure is shown in Figure 14.

The neural network was incorporated with PI and DZ models, so then the training algorithm could be implemented to update their weights in a real-time control experiment. At a frequency of 1 HZ, the number of Play operators selected for the classical PI model is 11, and the values of the operator thresholds and the weights of the operators are shown in Table 1.

In LabVIEW, the parameters shown in Table 1 are used to establish a classical PI model, and then the number of dead-zone operators is selected as 10; the values of the operator thresholds and the weights of the operators are shown in Table 2.

A modified Prandtl–Ishlinskii model was established in LabVIEW using the parameters shown in Table 1 and Table 2.

Firstly, the hysteresis nonlinearity characteristics of the piezoelectric stack actuator are measured without a hysteresis compensation control. Then, under the classic PI and DZ model, the hysteresis nonlinearity characteristics of the piezoelectric stack actuator are measured. Finally, the four-layer modified neural network with an adaptive learning model sets the Play operator weight update function and the dead-zone operator weight update function at the same time. The hysteresis nonlinearity characteristics of the piezoelectric stack actuator at different frequencies are verified under the above five conditions, and the hysteresis compensation ability of the model’s adaptive learning is analyzed.

#### 4.2.1. Hysteresis Compensation Without Model

**Figure 15:**
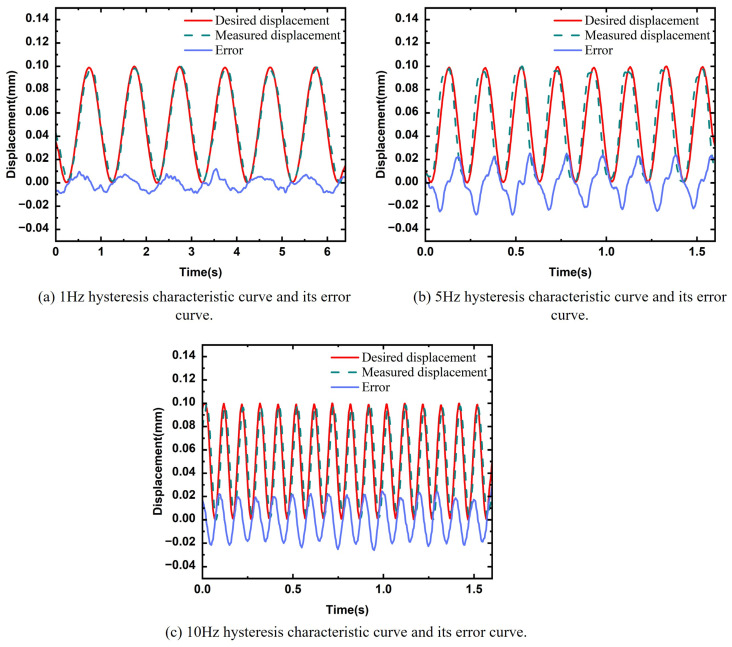
Hysteresis characteristic curve and error curve of piezoelectric stack actuator without control.

Without the hysteresis compensation control of the model, the hysteresis curves of the piezoelectric stack actuator at frequencies of 1 Hz, 5 Hz, and 10 Hz are respectively measured under the condition of the expected displacement signal of the sine wave. With time as the abscissa and displacement as the ordinate, the hysteretic nonlinear characteristic curves of the input and output of the piezoelectric stack actuator and the error curve are plotted, as shown in Figure 15a–c.

In observing the experimental results in Figure 15, it can be seen that the hysteresis characteristic curve of the piezoelectric stack actuator is related to the frequency of the de-driving voltage signal, and the larger the frequency of the driving voltage signal, the larger the hysteresis loop, the more obvious the hysteresis phenomenon is, and the larger the error value between the expected displacement signal and the measured displacement signal.

#### 4.2.2. Hysteresis Compensation Control of the Classic PI and DZ Model

**Figure 16:**
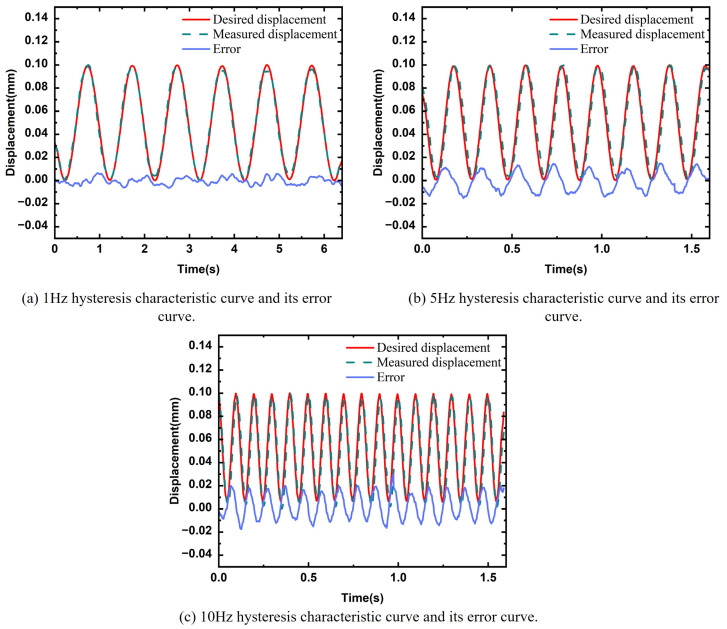
The hysteresis characteristic curve and error curve of the piezoelectric stack actuator under the classic PI and DZ model.

When the classic PI and DZ model was used to control the compensation, the hysteresis curves of the piezoelectric stack actuator at frequencies of 1 Hz, 5 Hz, and 10 Hz were measured. With time as the abscissa and displacement as the ordinate, the hysteretic nonlinear characteristic curves of the input and output of the piezoelectric stack actuator and the error curve are plotted, as shown in Figure 16a–c.

In observing the experimental results of Figure 16, it can be seen that under the control of the classic PI and DZ model, the hysteresis compensation effect of the piezoelectric stack actuator is particularly obvious at the frequency of 1 Hz. However, after 5 Hz, it is found that the hysteresis is still very obvious, and the model control effect is very insufficient.

#### 4.2.3. Experimental Verification of Four-Layer Modified Neural Network with Adaptive Learning Model

**Figure 17:**
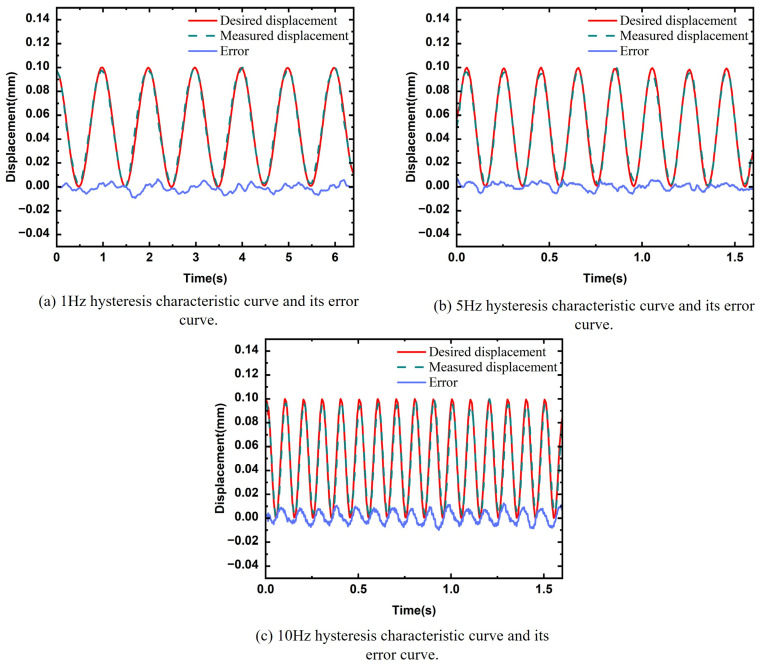
Hysteresis characteristic curve and error curve of four-layer modified neural network with adaptive learning model, $\alpha_{H}\;=\;0.0015,{\;\alpha }_{S}\;=\;0.0015$.

When $\alpha_{H}=0.0015,{\;\alpha }_{S}=0.0015$, the four-layer modified neural network with an adaptive learning model sets the Play operator weight to update the function in real time and the dead-zone operator weight to update the function in real time. The hysteresis curves of the piezoelectric stack actuator at frequencies of 1 Hz, 5 Hz, and 10 Hz are measured respectively. With time as the abscissa and displacement as the ordinate, the hysteretic nonlinear characteristic curves of the input and output of the piezoelectric stack actuator and the error curve are plotted, as shown in Figure 17a–c.

In observing the experimental results in Figure 17, it can be seen that after setting the real-time update function of the Play operator weight and the real-time update function of the dead-zone weight, the hysteresis compensation effect of the piezoelectric stack actuator in the frequency range of 1–10 Hz is more obvious.

**Figure 18:**
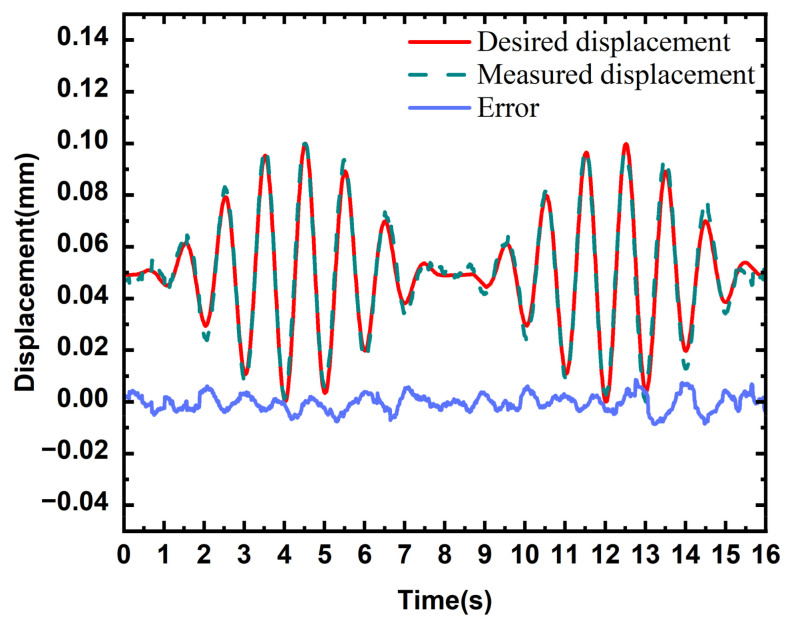
Hysteresis characteristic curve of 1 Hz amplitude and its error curve.

To further verify the performance of the controller under the non-periodic change signal, the experiment sets up a 1 Hz amplitude input signal, as shown in Figure 18. The results show that the controller exhibits a good tracking ability at 1 Hz.

### 4.3. Error Results Under Different Control Conditions

**Figure 19:**
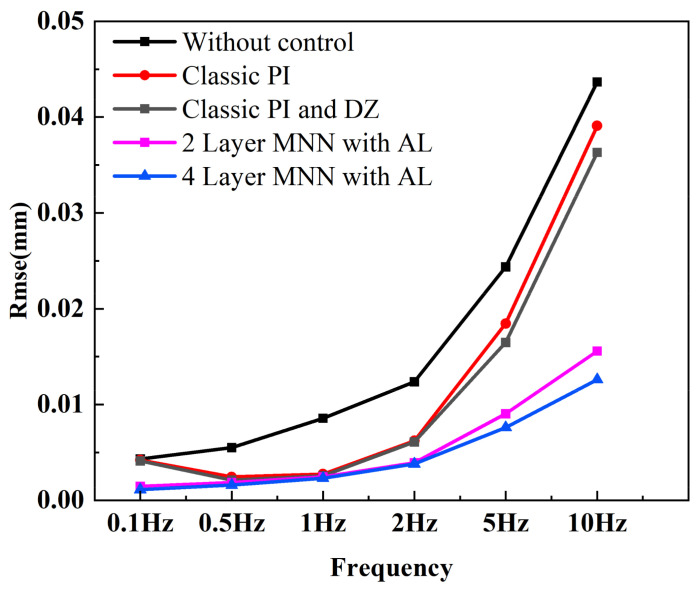
RMSE comparison of five control methods.

In order to further understand the reduction of the error value of piezoelectric stack actuator hysteresis compensation, it is necessary to compare the root mean square error (RMSE) results corresponding to different frequencies under the five control conditions. The root mean square error (RMSE) is calculated and listed in Table 3. This is shown in Figure 19.

According to the error results in Figure 19 (MNN with AL refers to the modified neural network with adaptive learning), when the classic PI and DZ model was added to the experimental platform for offline recognition, the RMSE was reduced from 0.008574 mm to 0.002745 mm at 1 Hz, which was a 67.98% reduction compared to the without control displacement error, and the compensation effect was significant.

After introducing adaptive learning to the four-layer modified neural network, the RMSE of the four-layer modified neural network with an adaptive learning model at 1 Hz is 0.002324 mm, which is 72.89% lower than that of the without controlled displacement error, and the compensation control effect is the most ideal. At the same time, because adaptive learning can update the weight coefficients of the four-layer modified neural network with an adaptive learning model in real time, the compensation control effect is better than that of the without control method and the classic PI and DZ model in the process of increasing the frequency to 10 Hz, even if the RMSE is 0.012607 mm at 10 Hz, which is 71.13% lower than that of the method with no control.

**Figure 20:**
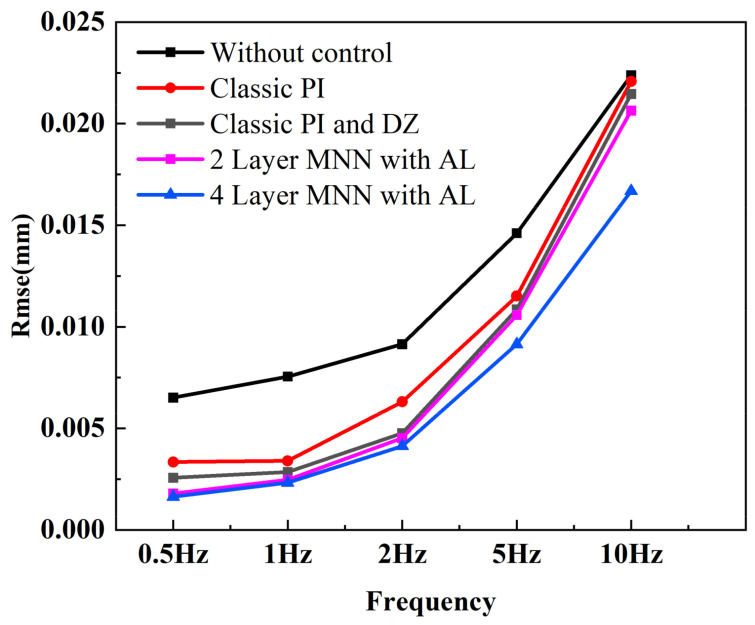
RMSE comparison of variable amplitude.

Under variable amplitude conditions, the root mean square error (RMSE) is calculated and listed in Table 4. This is shown in the Figure 20.

When the classic PI and DZ model was added to the experimental platform for offline identification, the RMSE was reduced from 0.007552 mm to 0.003398 mm at 1 Hz, which was 55% lower than the without control displacement error, and the compensation effect was more significant.

After introducing adaptive learning to the four-layer modified neural network, the RMSE of the four-layer modified neural network with an adaptive learning model at 1 Hz is 0.002422 mm, which is 67.93% lower than that of the without control displacement error, and the compensation control effect is the most ideal. At the same time, because adaptive learning can update the weight coefficient of the four-layer modified neural network with an adaptive learning model in real time, the compensation control effect is better than that of the without control method and the classic PI and DZ model in the process of increasing the frequency to 10 Hz, even if the RMSE is 0.016689 mm at 10 Hz, which is 25.44% lower than that of the without control method.

## 5. Conclusions

The experimental results show that the classic PI and DZ model reduces the RMSE by 67.98% at a frequency of 1 Hz compared to the without control condition, which can effectively reduce the hysteresis nonlinearity of the piezoelectric stack actuator and has a good hysteresis compensation effect. When the frequency increases, the hysteresis phenomenon of the piezoelectric stack actuator is still obvious, and the error is only reduced by 10.48% at 10 Hz. Contrasting the piezoelectric stack actuator’s performance between the classic PI and DZ model and the four-layer modified neural network with an adaptive learning model, the latter reduces the RMSE by 15.34% at the frequency of 1 Hz, and the error is reduced by 67.75% even at 10 Hz. Under the condition of variable amplitude, the piezoelectric stack actuator under the four-layer modified neural network with an adaptive learning model still has a good compensation effect. It is verified that the hysteresis nonlinearity control of the piezoelectric stack actuator has a good compensation control effect in the wide frequency band of 1~10 Hz based on a four-layer modified neural network with an adaptive learning model.

## Figures and Tables

**Table 1:** Values of Play operator thresholds and weights.

Threshold		Weights	
$r_{0}$	0	$W_{0}^{2}$	1.4606
$r_{1}$	0.05	$W_{1}^{2}$	−0.3575
$r_{2}$	0.10	$W_{2}^{2}$	−0.09663
$r_{3}$	0.15	$W_{3}^{2}$	−0.04099
$r_{4}$	0.20	$W_{4}^{2}$	−0.01023
$r_{5}$	0.25	$W_{5}^{2}$	−0.06621
$r_{6}$	0.30	$W_{6}^{2}$	0.04746
$r_{7}$	0.35	$W_{7}^{2}$	−0.04157
$r_{8}$	0.40	$W_{8}^{2}$	0.05142
$r_{9}$	0.45	$W_{9}^{2}$	−0.10397
$r_{10}$	0.50	$W_{10}^{2}$	0.15763

**Table 2:** Values of dead-zone operator thresholds and weights.

Threshold		Weights	
$R_{D0}$	0	$W_{0}^{4}$	1.02931
$R_{D1}$	0.1	$W_{1}^{4}$	0.0285291
$R_{D2}$	0.2	$W_{2}^{4}$	0.0202498
$R_{D3}$	0.3	$W_{3}^{4}$	−0.00158266
$R_{D4}$	0.4	$W_{4}^{4}$	−0.0329002
$R_{D5}$	0.5	$W_{5}^{4}$	−0.0607781
$R_{D6}$	0.6	$W_{6}^{4}$	−0.0489904
$R_{D7}$	0.7	$W_{7}^{4}$	−0.00125102
$R_{D8}$	0.8	$W_{8}^{4}$	0.052054
$R_{D9}$	0.9	$W_{9}^{4}$	0.0635702

**Table 3:** Comparison of five control methods.

Frequency	Without Control (mm)	Classic PI (mm)	Classic PI and DZ (mm)	2-Layer MNN with AL (mm)	4-Layer MNN with AL (mm)
0.1 Hz	0.004330	0.004215	0.004119	0.001450	0.001117
0.5 Hz	0.005498	0.002443	0.002101	0.001873	0.001594
1 Hz	0.008574	0.002745	0.002560	0.002464	0.002324
2 Hz	0.012376	0.006226	0.006099	0.003934	0.003809
5 Hz	0.024370	0.018447	0.016478	0.009036	0.007601
10 Hz	0.043665	0.039089	0.036319	0.015584	0.012607

**Table 4:** Comparison of five control methods of variable amplitudes.

Frequency	Without Control (mm)	Classic PI (mm)	Classic PI and DZ (mm)	2-Layer MNN with AL (mm)	4-Layer MNN with AL (mm)
0.5 Hz	0.006512	0.003341	0.002567	0.001796	0.001630
1 Hz	0.007552	0.003398	0.002854	0.002644	0.002422
2 Hz	0.009139	0.006307	0.004766	0.004519	0.004127
5 Hz	0.014610	0.011513	0.010857	0.010574	0.009141
10 Hz	0.022386	0.022082	0.021455	0.020636	0.016689

## Data Availability

The data that support the findings of this study are available from the corresponding author upon reasonable request.

## Abbreviations

The following abbreviations are used in this manuscript:

PI	Prandtl–Ishlinskii
MNN	Modified Neural Network
AL	Adaptive Learning
DZ	Dead Zone
